# An Unsupervised Framework for Online Spatiotemporal Detection of Activities of Daily Living by Hierarchical Activity Models

**DOI:** 10.3390/s19194237

**Published:** 2019-09-29

**Authors:** Farhood Negin, François Brémond

**Affiliations:** 1 INRIA, Sophia Antipolis, 2004 route des Lucioles, BP 93, 06902 Sophia Antipolis, France; 2 Institut Pascal, CNRS, UMR 6602, F-63171 Aubiere, France; 3 CoBTeK Team, Université Côte d’Azur, 06108 Nice, France; francois.bremond@inria.fr

**Keywords:** activity recognition, activity of daily living, assisted living, hierarchical activity models, unsupervised modeling

## Abstract

Automatic detection and analysis of human activities captured by various sensors (e.g., sequences of images captured by RGB camera) play an essential role in various research fields in order to understand the semantic content of a captured scene. The main focus of the earlier studies has been widely on supervised classification problem, where a label is assigned to a given short clip. Nevertheless, in real-world scenarios, such as in Activities of Daily Living (ADL), the challenge is to automatically browse long-term (days and weeks) stream of videos to identify segments with semantics corresponding to the model activities and their temporal boundaries. This paper proposes an unsupervised solution to address this problem by generating hierarchical models that combine global trajectory information with local dynamics of the human body. Global information helps in modeling the spatiotemporal evolution of long-term activities, hence, their spatial and temporal localization. Moreover, the local dynamic information incorporates complex local motion patterns of daily activities into the models. Our proposed method is evaluated using realistic datasets captured from observation rooms in hospitals and nursing homes. The experimental data on a variety of monitoring scenarios in hospital settings reveals how this framework can be exploited to provide timely diagnose and medical interventions for cognitive disorders, such as Alzheimer’s disease. The obtained results show that our framework is a promising attempt capable of generating activity models without any supervision.

## 1. Introduction

Activity detection has been considered as one of the major challenges in computer vision due to its utter importance in various applications including video perception, healthcare, surveillance, etc. For example, if a system could monitor human activities, it could prevent the elderly from missing their medication doses by learning their habitual patterns and daily routines. Unlike regular activities that usually occur in a closely controlled background (e.g., playing soccer), Activities of Daily Living (ADL) usually happen in uncontrolled and disarranged household or office environments, where the background is not a strong cue for recognition. In addition, ADLs are more challenging to detect and recognize, because of their unstructured and complex nature that create visually perplexing dynamics. Moreover, each person has his/her own ways to perform various daily tasks resulted in infinite variations of speed and style of performance which accordingly add extra complexity to detection and recognition tasks.

From the temporal aspect, detecting ADLs in untrimmed videos is a difficult task since they are temporally unconstrained and can occur at any time and in an arbitrarily long video (e.g., recordings of patients in a nursing home for days and weeks). Therefore, in activity detection, we are not only interested in knowing the types of the activities happening, but also we want to precisely know the temporal delineation of the activities in a given video (temporal activity localization).

Most of the available state-of-the-art approaches deal with this problem through detection by classification task [1,2,3]. These methods classify the generated temporal segments either in the form of sliding windows in multiple scales [4,5,6] or another external proposal mechanism [7,8]. These methods infer the occurring activity by exhaustively applying trained activity classifiers at each time segment. Although they achieve encouraging performances in short actions and small-scale datasets, these computationally expensive methods can not be applied conveniently to large-scale datasets and complex activities, such as ADLs. These methods are not capable of precisely predicting flexible activity boundaries. Temporal scale variability of the activities can be dealt with by using multiple-scale sliding window approaches, however, such methods are computationally expensive. To compensate the high computational cost of these methods, a class of methods [4,8,9] influenced by advancements in the field of object detection [10,11,12] have been developed in which instead of exhaustive scanning, perform a quick scan to single out candidate activity segments. The sought after activities are more likely to occur in these segments. In the second step, the activity classifiers are only applied to the candidate segments, therefore, remarkably reduce the operational cost. Although these methods have shown good results on activity recognition tasks [13,14,15], they rarely use context priors in their models. Another drawback is that instead of learning an end-to-end deep representation, they use off-the-shelf hand-crafted [16] or deep [17,18] representations independently learned from images. This will result in a poor detection performance as these representations are not intended and are not optimal for localization.

Most of the above-mentioned methods are single-layered supervised approaches. In the training phase of the activities, the labels are fully (supervised) [16,19,20] or partially (weakly supervised) [21,22] given. In other studies [23,24], the location of the person or the interacted object is known. Usually the discovery of temporal structure of activities is done by a linear dynamic system [25], a Hidden Markov Model [26], hierarchical grammars [27,28,29], or by spatiotemporal representation [30,31]. These methods have shown satisfying performances on well-clipped videos, however, ADLs consist of many simple actions forming a complex activity. Therefore, representation in supervised approaches is insufficient to model these activities and a training set of clipped videos for ADL cannot cover all the variations. In addition, since these methods require manually clipped videos, they can mostly follow an offline recognition scheme. There also exist unsupervised approaches [32,33] which are strong in finding meaningful spatiotemporal patterns of motion. Nevertheless, global motion patterns are not enough to obtain a precise classification of ADL. For long-term activities, many unsupervised approaches model global motion patterns and detect abnormal events by finding the trajectories that do not fit in the pattern [34,35]. Other methods have been applied to traffic surveillance videos to learn the regular traffic dynamics (e.g., cars passing a crossroad) and detect abnormal patterns (e.g., a pedestrian crossing the road) [36]. However, modeling only the global motion pattern in a single-layered architecture cannot capture the complex structure of long-term human activities. Moreover, a flat architecture focuses on one activity at a time and intrinsically ignores modeling of sub-activities. Hierarchical modeling, therefore, enables us to model activities considering their constituents in different resolutions and allows us to combine both global and local information to achieve a rich representation of activities.

In this work, we propose an unsupervised activity detection and recognition framework to model as well as evaluate daily living activities. Our method provides a comprehensive representation of activities by modeling both global motion and body motion of people. It utilizes a trajectory-based method to detect important regions in the environment by assigning higher priors to the regions with dense trajectory points. Using the determined scene regions, a sequence of primitive events can be created in order to localize activities in time and learn the global motion patterns of people. To describe an activity semantically, we can adapt a notion of resolution by dividing an activity into different granularity levels. This way, the generated models describe multi-resolution layers of activities by capturing their hierarchical structures and sub-activities. Hereupon, the system can move among different layers in the model to retrieve relevant information about the activities. We create the models to uniquely characterize the activities by deriving relative information and constructing a hierarchical structure. Additionally, a large variety of hand-crafted and deep features are employed as an implicit hint to enrich the representation of the activity models and finally perform accurate activity detection. To summarize, the core contributions of this paper set forth below:

- an unsupervised framework for scene modeling and activity discovery;
- dynamic length unsupervised temporal segmentation of videos;
- generating Hierarchical Activity Models using multiple spatial layers of abstraction;
- online detection of activities, as the videos are automatically clipped;
- finally, evaluating daily living activities, particularly in health care and early diagnosis of cognitive impairments.

Following these objectives, we conducted extensive experiments on both public and private datasets and achieved promising results. The rest of the paper is organized as follows: Section 2 presents the related studies from the literature. Section 3 explains our suggested approach followed by describing conducted experiments in Section 4. Lastly, Section 5 concludes the paper.

## 2. Related Work

### 2.1. Activity Recognition

For the past few decades, activity recognition has been extensively studied and most of the proposed methods are supervised approaches based on the hand-crafted perceptive features [16,17,20,21,22,23,37,38]. The linear models [25,26,39,40] gain the most popularity through modeling action transitions. Later on, more complicated methods modeling activity’s hierarchical and graphical relations were introduced [28,29,41].

Recent re-emergence of deep learning methods has been led to remarkable performances in various tasks. That success followed by adapting convolutional networks (CNNs) to activity recognition problem for the first time in [42]. The inclination toward using CNNs in the field reinforced by introduction of two-stream [43] and 3D-CNN [17] architectures to utilize both motion and appearance features. Most of these methods are segment-based and usually use a simple method for aggregating the votes of each segment (frame or snippet). There are also other approaches that investigate long-range temporal relations of activities through temporal pooling [37,44,45]. However, the main assumptions in these methods are that the given videos should be manually clipped and the activities should take place in the entire duration of the videos. Therefore, the temporal localization of those activities is not taken into account.

### 2.2. Temporal and Spatiotemporal Activity Detection

The goal in activity detection is to find both the beginning and end of the activities in long-term untrimmed videos. The previous studies performed in activity detection were mostly dominated by sliding window approaches, where the videos are segmented by sliding a detection window followed by training classifiers on various feature types [4,6,46,47,48]. These methods are computationally expensive and produce noisy detection performances, especially in activity boundaries.

Recently, several studies [4,9,49,50] incorporate deep networks and try to avoid the sliding window approach and search for activities with dynamic lengths. This is usually achieved by temporal modeling of activities using Recurrent Neural Network (RNN) or Long Short-Term Memory (LSTM) networks [51,52]. For example, [9] uses an LSTM to encode Convolution3D (C3D) [17] features of each segment and classifies it without requiring an extra step for producing proposals. However, their model is still dependant on hand-crafted features. In order to resolve the problem of short dependencies in RNN based methods, time-series models, such as Temporal Convolutional Networks (TCN) [53,54], employ a combination of temporal convolutional filters and upsampling operations for acquiring long-range activity relations. However, applying convolutional operations on the local neighborhood for detecting long-range dependencies is not efficient in terms of computational time. Moreover, many methods use the concept of Actioness [55] to produce initial temporal activity proposals. Actioness indicates the likelihood of a generic activity localized in the temporal domain. Reliability of the Actioness hinges upon the correctness of distinguishing the background. Unlike conventional activity datasets which contain many background segments, long activities in ADL datasets are usually linked through short background intervals. Accordingly, methods [2,56] which are relied on Actioness cannot effectively determine the temporal boundary of ADLs in such datasets.

The methods used in [57,58,59,60,61] explore the videos to detect activities in spatial and temporal domains simultaneously. Some methods [61,62] employ a supervoxel approach to perform spatiotemporal detection, while others use human detectors [60,63] and treat the detection problem as a tracking problem [57,59]. Most of these approaches require object detection for a more accurate detection and therefore, demand exhaustive annotation of objects in long videos which is a tedious and time-consuming process. Note that the activity detection problem is closely related to object detection problem from images. A major part of the studies in the literature is inspired by object detection, but as it is not the focus of this study, we do not review object detection based methods here. It is worth mentioning that even though the models currently do not utilize object detection features, they still have a flexible design which depends on the availability of the features, any number and types of features can be included or excluded from the models.

Apart from the supervised methods mentioned above, recently there has been increasing attention on methods with unsupervised learning of activities. A pioneer study conducted by Guerra-Filho and Aloimonos [64] sought to overcome the problem of temporal segmentation of human motion which does not require training data. They suggest a basic segmentation method followed by clustering step relied on motion data. Based upon these motion descriptors, they make use of a parallel synchronous grammar system to learn sub-activities of a long activity analogous to identify words in a complete sentence. Another study performed by Fox et al. [65] makes use of the non-parametric Bayesian approach to model pattern of several related atomic elements of an activity identical to elements of a time series without any supervision. Similarly, Emonet et al. [66] proposes an unsupervised Non-parametric Bayesian methods based on Hierarchical Dirichlet Process (HDP) to discover recurrent temporal patterns of words (Motifs). Their method automatically finds the number of topics, recurrence of the activities and the time of their occurrence. Furthermore, several methods take advantage of temporal structure of video data for adjusting parameters of deep networks without using any labeled data for training [67,68]. Some others [69,70,71,72] utilize temporal pattern of activities in an unsupervised way for representation, hence, for detection of activities. Lee et al. [71] formulates representation learning as a sequence sorting problem by exploiting the temporal coherence as a supervisory hint. Temporally shuffled sequence of frames are taken as input for training a convolutional neural network to determine the correct order of the shuffled sequences. In another study conducted by Ramanathan et al. [72], a ranking loss based approach is presented for incorporating temporal context embedding based on past and subsequent frames. A data augmentation technique is also developed to emphasize the effect of visual diversity of context embedding. Fernando et al. [70] leverages the parameters of a frame ranking function as a new video representation method to encode temporal evolution of activities in the videos. The new representation provides a latent space for each video where a principled learning technique is used to model activities without requiring annotation of atomic activity units. Similarly, [73] encodes structured representation of postures and their temporal evolution as motion descriptors for activities. A combinatorial sequence matching method is proposed to realize the relationship between the frames and a CNN is also utilized to detect the conflict of transitions.

So far, state-of-the-art methods are constrained by full supervision and require costly frame level annotation or at least an ordered list of activities in untrimmed videos. By growing the size of the video datasets, it is very important to discover activities in long untrimmed videos. Therefore, recent works propose unsupervised approaches to tackle the problem of activity detection in untrimmed videos. In this work, we use training videos to specify temporal clusters of segments that contain similar semantics throughout the all training instances.

## 3. Unsupervised Activity Detection Framework

The proposed framework provides a complete representation of human activities by incorporating (global and local) motion and appearance information. It automatically finds important regions in the scene and creates a sequence of primitive events in order to localize activities in time and to learn the global motion pattern of people. To perform accurate activity recognition, it uses a large variety of features, such as Histogram of Oriented Gradients (HOG), Histogram of Optical Flow (HOF), or deep features, as an implicit hint.

**Figure 1:**
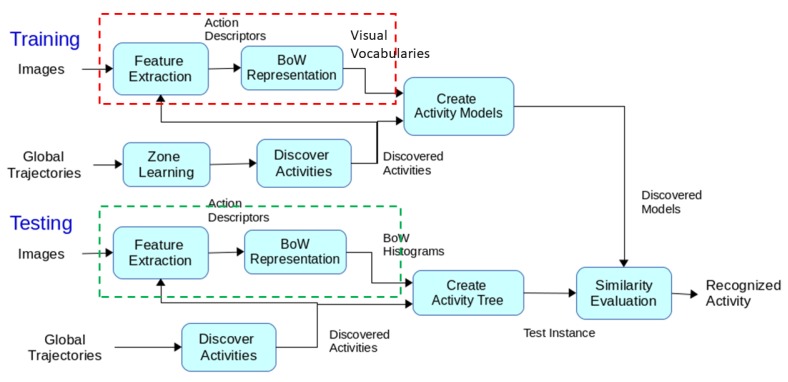
The flow diagram of the unsupervised framework: Training and Testing phases. The **red dashed box** shows the training of the visual codebooks of the descriptors. The **green box** in the testing phase shows the descriptor matching procedure.The flow diagram of the unsupervised framework.

As Figure 1 shows, first, long-term videos are processed to obtain trajectory information of the people’s movement (input). This information is used to learn scene regions by finding the parts of the scene with a higher prior for activities to occur, i.e., dense regions in terms of trajectory points. A common approach is to assume that there is only one kind of action occurs inside a region [34,36,74]. However, in unstructured scene settings, this assumption may not be valid. In order to distinguish actions occurring inside the same region, we benefit from the local motion and appearance features (visual vocabularies). The learned regions are employed to create primitive events which basically determine primitive state transitions between adjacent trajectory points. Based on the acquired primitive events, a sequence of discovered (i.e., detected) activities is created to define the global motion pattern of people, such as staying inside a region or moving between regions. For each discovered activity, motion statistics, such as time duration, etc., are calculated to represent the global motion of the person. Finally, a model of a certain activity is constructed through the integration of all extracted features and attributes. During the testing phase, the learned regions are used to obtain primitive events of the test video. Again, the video is clipped using discovered zones and the action descriptors are extracted for each discovered activity. Similar to the training phase, for each discovered activity, by combining the local motion information with global motion and other attributes, an activity model is constructed. To recognize activities, a comparison is performed between trained activity models and acquired test activity. A similarity score between the test instance and trained activity models are calculated by comparing global and local motion information of the models. Finally, the activity model with the maximum similarity score is considered as recognized activity of the test instance. Through all the steps, an online scheme is followed to perform continuous activity detection in assisted living scenarios. The subsequent sections describe different parts of the framework in more detail.

### 3.1. Feature Extraction

For local feature detection, improved dense trajectories [75] are employed which densely sample points of interests and track them in consecutive frames of a video sequence. The points of interests are sampled using a *W* pixels sized grid in multiple scales. Each trajectory is tracked separately at each scale for *L* frames and the trajectories exceeding this limit are removed from the process. Once the trajectories are extracted, the descriptors in the local neighborhood of the interest-points are computed. There are three different types of descriptors extracted from the interest-points: Trajectory shape, motion (HOF and Motion Boundaries Histogram, a.k.a MBH [75]), and appearance (HOG [76]) descriptors.

Given a trajectory of length *L*, its shape can be described by a sequence ($S=(∆P_{t},\cdots ,∆P_{t+L-1})$) of displacement vectors: $∆P=(P_{t+1}-P_{t})$. The final descriptor (trajectory shape descriptor, a.k.a TSD) is computed by normalizing the magnitude of the displacement vector. Other than spatial scales, the trajectories are also calculated in multiple temporal scales in order to represent actions that rapidly occurred.

Motion descriptors (HOF and MBH) are computed in a volume around the detected interest-points and throughout their trajectories (spatiotemporal volume). Size of the constructed volume is N×N pixels around the interest-point and *L* frames long. For all of the grids in the spatiotemporal volume, the descriptors are calculated and concatenated to represent the final descriptor. While motion-based descriptors focus on the representation of the local motion, appearance descriptor (HOG) represents static appearance information by calculating gradient vectors around the calculated trajectory points.

Geometrical descriptors are also used for representing the spatial configuration of the skeleton joint information and model human body pose in each frame. To represent the skeleton, both joints’ Euclidean distances and angles in polar coordinate are calculated using normalized joint positions. In order to preserve temporal information in pose representation, a feature extraction scheme based on temporal sliding window is adapted [77]. At each time instance, Euclidean distances between all the joints are calculated. Besides, for each joint, distance from other instances’ joints included in the sliding window is calculated and stored. If $J_{i}^{t}$ represents features of joint *i* at time *t* and *w* shows the sliding window size: $J_{i}^{t}=\left[x_{i}^{t},y_{i}^{t}\right]$ defines raw skeleton features at time *t*, where i=1,…,8. Then, $F^{d}$ calculates the distance descriptor: $F^{d}=\sqrt{{\left(x_{i}^{t}-x_{j}^{t^{\prime }}\right)}^{2}+{\left(y_{i}^{t}-y_{j}^{t^{\prime }}\right)}^{2}}$. Similarly, to calculate angular feature in polar coordinate, we use: $F^{a}=\arctan\left(x_{i}^{t}-x_{j}^{t^{\prime }},y_{i}^{t}-y_{j}^{t^{\prime }}\right)$, where $t^{\prime }\in \left\{t,t-1,\ldots ,t-w\right\},t^{\prime }>0$ and i,j=1,2,…,8 for both equations. Combining these features produces the final descriptor vector $F=[F^{d},F^{a}]$.

In order to compare the effect of hand-crafted and deep features on our generated activity models, the framework uses Trajectory-Pooled Deep-Convolutional Descriptors (TDD) introduced in [37]. Computing these features are similar to dense trajectory descriptors. The main difference here is that rather than computing the hand-crafted features around the spatiotemporal volume of the trajectories, deep features are extracted using Convolutional Neural Network (CNN) maps. To compute these features, multi-scale convolutional feature maps pool deep features around the interest-points of the detected trajectories. The two-stream ConvNet architecture proposed by Simonyan [43] is adapted for TDD feature extraction. The two-stream CNN consists of two separate CNNs: spatial and temporal networks. The motion features (temporal) are trained on optical flow and extracted using conv3 and conv4 layers of CNN. Additionally, for the training of the appearance features (spatial) on RGB frames, conv4 and conv5 layers of CNN are used.

### 3.2. Global Tracker

Information about the global position of the subjects is indispensable in order to achieve an understanding of long-term activities. For person detection, the algorithm in [78] is applied that detects head and shoulders from RGBD images. Trajectories of the detected people in the scene are obtained using the multi-feature algorithm in [79] using 2D size, 3D displacement, color histogram, the dominant color, and covariance descriptors as a feature and the Hungarian algorithm [80] to maximize the reliability of the trajectories. We use the control algorithm in [81] to tune tracking parameters in an online manner. The output of the tracking algorithm is the input for the framework:

(1) $$Input=\left\{Seq_{1},\ldots ,Seq_{n}\right\},$$

where $Seq_{i}=Traj_{1},\cdots ,Traj_{T}$. *i* is the label of the tracked subject and *T* is the number of trajectories in each sequence. Each scene region characterizes a spatial part of the scene and will be represented as a Gaussian distribution: $SR_{i}∼\left(\mu^{i},\sigma^{i}\right)$.

### 3.3. Scene Model

In most of the trajectory-based activity recognition methods, a priori contextual information is ignored while modeling the activities. The proposed framework performs automatic learning of the meaningful scene regions (topologies) by taking into account the subject trajectories. The regions are learned at multiple resolutions. By tailoring topologies at different levels of resolution, a hierarchical scene model is created. A topology at level *l* is defined as a set of scene regions (SR):

(2) $$T_{level_{l}}=\left\{SR_{0},\ldots ,SR_{k-1}\right\}.$$

*k* indicates the number of scene regions defining the resolution of the topology. The scene regions are obtained through clustering which takes place in two stages. This two stages clustering helps to reduce the effect of outlier trajectory points in the overall structure of the topologies. In the first stage, the interesting regions for each subject in the training set are found by clustering their trajectory points. For each Seq, the clustering algorithm produces *k* clusters: $Cluster\left(Seq_{i}\right)=\left\{Cl_{1},\ldots ,Cl_{k}\right\}$ where each resulted cluster characterizes the scene based on the motion information of subject *i*. μ and ω parameters of the distribution of the $SR_{i}$ are calculated from the clustering. *C*th cluster center ($Cl_{c}$) corresponds to scene region *i* ($SR_{i}$). For $SR_{i}$, μ is the spatial coordinate of the cluster centroid: $SR_{i}\left(\mu \right)=centroid\left(Cl_{c}\right)$ and the standard deviation σ is computed from the point coordinate sequence of the trajectory set. The second stage of the clustering merges individual scene regions into a single comprehensive set of regions. Each region is a new cluster (Cl) in the second stage partitioning the obtained cluster centroids in the first stage. K-means algorithm is used for the clustering where the optimal value of *K* is calculated based on the Bayesian Information Criterion (BIC) [82]. We define a scene model as a set of scene regions (topologies) at different resolutions:

(3) $$SceneModel=<Topology_{\mathrm{highlevel}},Topology_{\mathrm{midlevel}},Topology_{\mathrm{lowlevel}}>.$$

**Figure 2:**
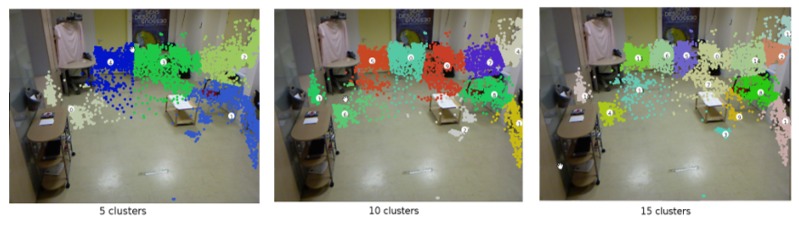
Example of K-means clustering using city-block distance measurements of Centre Hospitalier Universitaire de Nice (CHU) dataset. The number of clusters is set to 5, 10, and 15.

We create a model with topologies at three levels, each aims to describe the scene at a high, medium and low degree of abstraction. Figure 2 depicts an example of the calculated scene regions in a hospital room in Centre Hospitalier Universitaire de Nice (CHU) dataset (https://team.inria.fr/stars/demcare-chu-dataset/).

### 3.4. Primitive Events

To fill the gap between the low-level image features and high-level semantic description of the scene, an intermediate block capable of linking the two is required. Here, we describe a method that defines a construction block for learning the activity models. With a deeper look at the activity generation process, it can be inferred that the abstraction of low-level features into high-level descriptions does not happen in a single step and this transition is gradual. As a solution, we use an intermediate representation named Primitive Event (PE). Given the two consecutive trajectory data points ($Traj_{i}$ and $Traj_{j}$), by using their distance from the cluster centroids, their corresponding scene regions (StartRegion and EndRegion) can be found. A primitive event is represented as a pair of directed scene regions of these trajectory points:

(4) $$PrimitiveEvent=\left(StartRegion\to EndRegion\right),$$

where *StartRegion* and *EndRegion* variables take values of SR indices. For example, if *StartRegion* of $Traj_{i}$: $SR_{2}$ and *EndRegion* of $Traj_{j}$: $SR_{4}$ then, we will have (2→4) as a primitive event. PE describes an atomic motion block and is used for characterizing motion of a person in a scene. This way, a whole sequence of trajectory can be translated into PEs. A *Primitive Event*’s type is *Stay*, when the region labels (Such as SR1) stay constant between two time intervals. It is equivalent to a sequence of *Stay*s in the scene region *P*:

(5) $$Primitive\;Event=Stay_{P\_P}.$$

When a *Primitive Event*’s type is *Change*, a change of region (from region *P* to region *Q*) between two successive time instants (i.e., two successive trajectory points) occurs. It is equivalent to a region transition:

(6) $$Primitive\;Event=Change_{P\_Q}.$$

The duration of the current status (stay/change) can be calculated simply by $Duration=\frac{EndEventFrame-BeginEventFrame}{fps}$, where fps is the frame rate of the recorded images. Using a learned topology *T* for every video sequence, a corresponding primitive event sequence $PE_{seq}$ is calculated:

(7) $$PE_{seq}=\left(<PE_{1},\cdots ,PE_{n}>,T\right).$$

A primitive event sequence provides information regarding the underlying structure of long-term activities.

### 3.5. Activity Discovery (Detection)

We refer to the detection of the boundaries of the activities as *Activity Discovery*. Annotating the beginning and end of the activities is a challenging task even for humans. The start/end time of the annotated activities varies from one human annotator to another. The problem is that humans tend to pay attention to one resolution at a time. For example, when a person is sitting on a chair, the annotated label is “sitting“. Later, when the subject “moves an arm“, she is still sitting. Discovering activities using a different resolution of the trained typologies helps to automatically detect these activity parts and sub-parts at different levels of activity hierarchy using previously created semantic blocks (Primitive Events). Input for activity discovery process is a spatiotemporal sequence of activities described by primitive events. After the activity discovery process: (1) The beginning and end of all activities in a video are estimated and the video is automatically clipped. (2) The video is classified naively into discovered activities indicating similar activities in the timeline. A discovered activity (DA) is considered either as (1) staying in current state (“*Stay*“) or (2) changing of the current state (“*Change*“). Basically, a *Stay* pattern is an activity that occurs inside a single scene region and is composed of primitive events with the same type:

(8) $$Discovered\;Activity=Stay_{P\to P}=\left\{Stay\;PEs\right\}.$$

A “*Change*” pattern is an activity that happens between two topology regions. A “*Change*” activity consists of a single primitive event of the same type:

(9) $$Discovered\;Activity=Change_{P\to Q}=Change\;PE.$$

**Figure 3:**
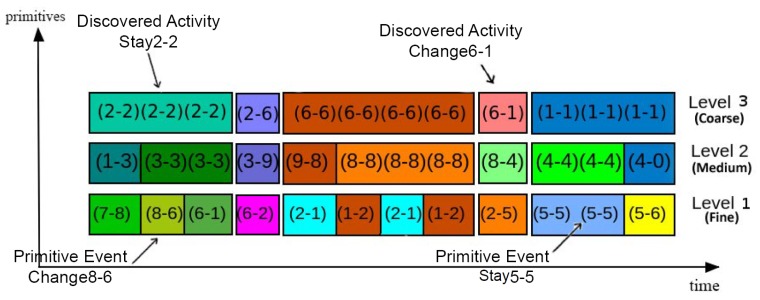
A sample video encoded with primitive events and discovered activities in three resolution levels.

Although detection of primitive events takes place at three different resolutions, the activity discovery process only considers the coarse resolution. Therefore, after discovery process, the output of the algorithm for the input sequence is a data structure containing information about the segmented input sequence in the coarse level and its primitive events in two other lower levels. This data structure holds spatiotemporal information similar to the structure in Figure 3. The algorithm for this process simply checks for primitives’ boundaries and constructs the data structure for each discovered activity. Employing DAs and PEs, it shows the hierarchical structure of an activity and its sub-activities.

Although Discovered Activities present global information about the movement of people, it is not sufficient to distinguish activities occurring in the same region. Thus, for each discovered activity, body motion information is incorporated by extracting motion descriptors (Section 3.1). These descriptors are extracted in a volume of *NxN* pixels and *L* frames from videos. Fisher Vector (FV) method [83] is then followed to obtain a discriminative representation of activities. The descriptors are extracted for all Discovered Activities that are automatically computed. The local descriptor information is extracted only for Discovered Activities at the coarse resolution level.

### 3.6. Activity Modeling

Here, the goal is to create activity models with high discriminative strength and less susceptibility to noise. We use attributes of an activity and its sub-activities for modeling and accordingly, learning is performed automatically using the DAs and PEs in different resolutions. Learning such models enables the algorithm to measure the similarity between them. To create the models, a method for assembling the DAs and PEs from different resolutions is required. This is achieved by the concept of hierarchical neighborhood.

#### 3.6.1. Hierarchical Neighborhood

The hierarchical representation of activity *A* at resolution level *l* is a recursive representation of the links between *A* and its primitive events $B_{i}$ at the finer resolutions:

(10) $$A_{neighborhood}=\left(\left(B1,B1_{neighborhood}\right),\cdots ,\left(Bn,Bn_{neighborhood}\right)\right).$$

B1,⋯,Bn are the primitive events of *A* in the next finer resolution. The links between the different levels are established using temporal overlap information. For example, primitive event *B* is sub-activity of activity *A* in a higher level if their temporal interval overlaps in the activity timeline. Formally, *B* is sub-activity of *A* if the following statement holds:

(11) $$\begin{array}{c} \left(\left(startFrame_{A}\leq startframeB\right)\wedge \left(endFrame_{A}\geq startFrame_{B}\right)\right)\\ \left\|(\left(startFrame_{A}\leq endFrame_{B}\right)\wedge \left(endFrame_{A}\geq endFrame_{B}\right)\right)\\ \left\|(\left(startFrame_{A}\leq startFrame_{B}\right)\wedge \left(endFrame_{A}\geq endFrame_{B}\right)\right)\\ \|(\left(startFrame_{A}\geq startFrame_{B}\right)\wedge \left(endFrame_{A}\leq endFrame_{B}\right)). \end{array}$$

By applying (10) to a discovered activity, we can find the primitives in its neighborhood. This automatic retrieval and representation of the neighborhood of a DA help in creating the hierarchical activity models.

#### 3.6.2. Hierarchical Activity Models

**Figure 4:**
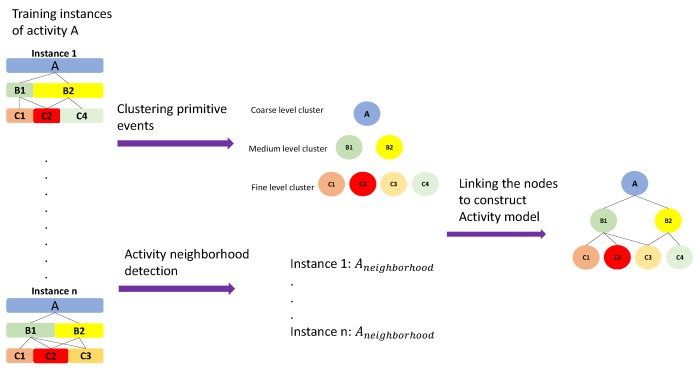
The process of creating activity tree. The Primitive Events (PEs) from the training instances are clustered into nodes and, at the same time, the neighborhood set is detected. The final structure is constructed with those building blocks.

Hierarchical activity model (HAM) is defined as a tree that captures the hierarchical structure of daily living activities by taking advantage of the hierarchical neighborhoods to associate different levels. For an input DA ($A_{neighborhood}$) and its neighborhood, the goal is to group similar PEs obtained by clustering to create nodes (*N*) of the activity tree. Clustering is performed using *Type* attribute of the PEs which groups PEs of the same type in one cluster. This process is repeated for all levels. After clustering, nodes of the tree model are determined followed by linking them together to construct the hierarchical model of the tree. The links between the nodes are realized from the activity neighborhood of each node (Figure 4 shows the complete procedure of creating an activity tree from neighborhood set instances of a DA). After linking, a complete tree structure of the given DA is obtained and the model is completed by adding attribute information for nodes of the tree. Each node in the activity tree contains information about the similar detected primitive events sharing similar properties, such as duration and type of the primitive, as well as similar sub-activities in the lower level. So, a node is the representative of all the similar primitives in that level. Each node has two types of properties. The node attributes that store information about primitive events such as average duration of its constituents as well as information about parent node and the associated nodes in the lower level of the hierarchy. The nodes can keep different spatial and temporal attributes about the activity and its sub-activities. The former type consists of:

- *Type attribute* is extracted from the underlying primitive or discovered activity (in case of the root node). For node *N*, $Type_{N}=Type_{PE}\;or\;Type_{DA}$, where Type of PEs and DAs are either *Stay* or *Change* states.
- *Instances* list PEs of training instances indicating the frequency of each PE included in the node.
- *Duration* is a Gaussian distribution $Duration(\mu_{d},\sigma_{d}^{2})$ describing the temporal duration of the PEs ($\left\{PE_{1},PE_{2},\cdots ,PE_{n},\right\}$) or discovered activities ($\left\{DA_{1},DA_{2},\cdots ,DA_{n},\right\}$) of the node. It is frame length of the primitives or discovered activities calculated as:
  (12) $$\mu_{d}=\sum_{i,j=1}^{n}\frac{\left(endframe_{PE_{i}orDA_{j}}-startframe_{PE_{i}orDA_{j}}\right)}{n},$$
  (13) $$\sigma_{d}^{2}=E\left[{\left(\left(endframe_{PE_{i}orDA_{j}}-startframe_{PE_{i}orDA_{j}}\right)-\mu_{d}\right)}^{2}\right],$$
  where *n* is the number of PEs or DAs.
- *Image Features* store different features extracted from the discovered activities. There is no limitation on the type of feature. It can be extracted hand-crafted features, geometrical or deep features (Section 3.1). It is calculated as the histogram of the features of the instances in the training set.
- *Node association* indicates the parent node of the current node (if it is not the root node) and the list of neighborhood nodes in the lower levels.

**Figure 5:**
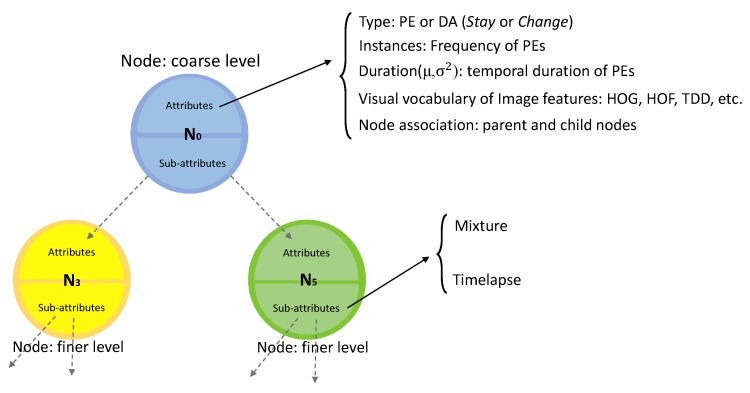
An example of model architecture in node level where each node is composed of attributes and sub-attributes. DA = discovered activity; HOG = istogram of Oriented Gradients; HOF = Histogram of Optical Flow; TDD = Trajectory-Pooled Deep-Convolutional Descriptors.

The above-mentioned attributes do not describe the relationship between the nodes which is important in the overall description of the activities. In order to model the relationship among the nodes, for each node, two other attributes are defined regarding their sub-nodes: *Mixture* and *Timelapse*. *Mixture* shows contribution of the type of the sub-activities ($Stay_{2-2}$) in the total composition of sub-nodes. This number is modeled with a Gaussian mixture $\Theta_{type}^{mixture}$. *Timelapse* of the nodes (with the same type and level in different training instances) represents the distribution of the temporal duration of the sub-nodes. This attribute is also computed as a Gaussian distribution $\Theta_{type}^{timelapse}$. The created HAM structure is a hierarchical tree that provides recursive capabilities. Accordingly, it makes the calculation of the attributes and the score in the recognition step efficient and recursive. Figure 5 illustrates an example of a HAM model with the nodes and their corresponding attributes and sub-attributes.

### 3.7. Descriptor Matching of Tree Nodes

Descriptor matching can be denoted as a method that captures the similarity between a given local dynamic information of an activity and a set of calculated multi-dimensional distributions. The obtained descriptor vectors (*H*) characterize local motion and appearance of a subject. Knowing the vector representation of the descriptors of discovered activities enables the use of a distance (Equation (14)) measurement to characterize the similarity between different activities.

**Figure 6:**
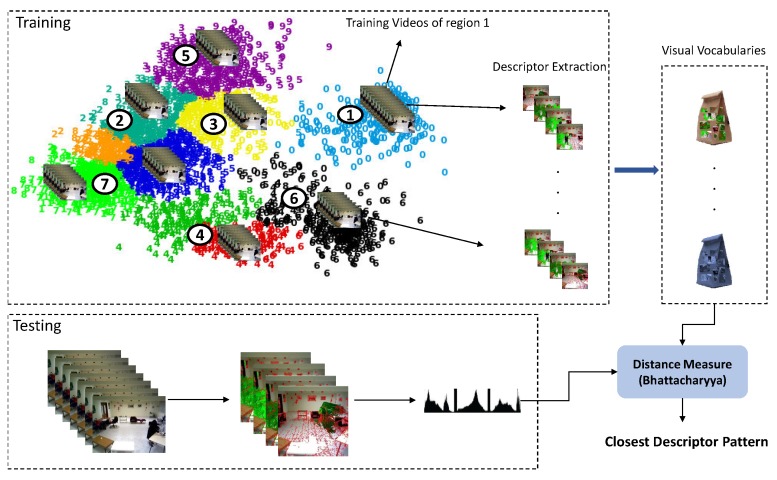
The process of learning visual codebook for each activity model and matching the given activity’s features with the most similar dictionary: Training and Testing phases.

As it is shown in Figure 6, in training, the scene model is used to clip the long videos to the short clips belonging to each region. Next, the descriptors of the clipped videos are extracted and employed to learn a visual codebook *V* (one for each region) by clustering the descriptors (using k-means). The codebook of each region is stored in the created activity model of that region. During the testing phase, when a new video is detected by the scene model, its descriptors are extracted and the feature vectors are created. These feature vectors are encoded with the learned dictionaries of the models. The distance of the current descriptor is calculated with the trained codebooks of all regions (to find the closest one) using the Bhattacharyya distance:

(14) $$Distance\left(H,V\right)=\sum_{i=1}^{N}BC\left(H,V_{i}\right),$$

where *N* is the number of learned code words and BC is the Bhattacharyya coefficient:

(15) $$BC=\sum_{x,y=1}^{N,M}H\left(x\right)V_{i}\left(y\right).$$

*N* and *M* display dimensions of the descriptor and trained codebooks, respectively. The most similar codebook is determined by the minimum distance score acquired. That codebook (and its corresponding activity model) is assigned by a higher score in the calculation of the final similarity score with the test instance in the recognition phase.

### 3.8. Model Matching for Recognition

To measure the similarity among the trained HAM models, different criteria can be considered. The assumed criterion can vary from one application to another. While one application can emphasize more on the duration of activities, local motion can be more important for others. Although these criteria can be set depending on the application, the weights of the feature types are learned to determine the importance of each type. The recognition is carried out in five steps as follows:

1. Perceptual information, such as trajectories of a new subject, is retrieved.
2. Using the previously learned scene model, the primitive events for the new video are calculated.
3. By means of retrieved primitive events, the discovered activities are calculated.
4. Using the collected attribute information, a test instance HAM ($\omega^{*}$) is created.
5. The similarity score of the created HAM and trained HAM models are calculated and the activity with the highest score is selected as the target activity.

Once the activity models are trained, to find the one that matches with an activity in a test video, we follow a Bayesian scheme. We choose the final label using the Maximum A Posteriori (MAP) decision rule. If $\Omega =\{\omega_{1},\ldots ,\omega_{S}\}$, where S=|Ω| represent the set of generated activity models and given the data for an observed test video, $\omega^{⋆}$, we select the activity model, $\omega_{i}$, that maximizes the likelihood function (Equation (16)):

(16) $$p\left(\omega^{⋆}|\omega_{i}\right)=\frac{p\left(\omega^{⋆}\right)p\left(\omega_{i}|\omega^{⋆}\right)}{p\left(\omega_{i}\right)},$$

where $p\left(\omega_{i}|\omega^{⋆}\right)$ denotes the likelihood function defined for activity models $\omega_{1},\ldots ,\omega_{s}$ in model set Ω. We assume that the activity models are independent. Therefore, *a priori* probability of trained models $p\left(\omega_{1},\ldots ,\omega_{s}\right)$ is considered equal. We can eliminate $p\left(\omega_{i}\right)$ and use the following formula (Equation (17))

(17) $$\tilde{p}\left(\omega^{⋆}|\omega_{i}\right)=p\left(\omega^{⋆}\right)\prod_{i=1}^{S}p\left(\omega_{i}|\omega^{⋆}\right).$$

$p\left(\omega^{⋆}\right)$ is the relative frequency of $\omega^{⋆}$ in the training set. Since the generated models are constructed following a tree structure, the likelihood value should be calculated recursively to cover all nodes of the tree. For each model, the recursive probability value is, therefore, calculated as Equation (18):

(18) $$p\left(\omega_{i}|\omega^{⋆}\right)=p\left(\omega_{i}^{\left[l\right]}|\omega^{⋆[l]}\right)+Recur\left(\left[l\right]-1\right).$$

Recur recursively calculates the probabilities of the nodes in lower levels and stops when there is no more leaf to be compared. Superscripts index the levels of the tree ([*l*] = 1, 2, 3). $p(\omega_{i}^{\left[l\right]}|\omega^{⋆[l]})$ calculates probability in the current node given $\omega^{⋆}$ and $p(\omega_{i}^{\left[l\right]}|\omega^{⋆[l-1]})$ returns the probability values of this node’s child nodes (sub-activities). Given the data for node *n* of the activity in the test video, $\omega^{⋆}\left(n\right)=\{type^{⋆}\left(n\right),$ $duration^{⋆}\left(n\right),$ $l^{⋆}\left(n\right)\}$ and the activity model *i*, $\omega_{i}\left(n\right)=\{type^{i}\left(n\right),$ ${∆}_{duration}^{i}\left(n\right),$ $Distance^{i}\left(n\right)\}$, where ${∆}_{duration}^{i}=\left\{\mu^{i},\sigma^{i}\right\}$. The likelihood function for node *n* is defined as Equation (19).

(19) $$\begin{array}{cc} \tilde{p}\left(\omega_{i}{\left(n\right)}^{l}|\omega^{⋆}\left(n\right)\right)= & p\left(\omega^{⋆}\left(n\right)|type^{⋆}=type^{i}\left(n\right)\right)*\\ & p\left(duration^{⋆}\left(n\right)|{∆}_{duration}^{i}\left(n\right)\right)*\\ & p\left(\omega_{⋆}\left(n\right)|l^{⋆}=Distance^{i}\left(n\right)\right). \end{array}$$

$p\left(\omega^{⋆}\left(n\right)|type^{⋆}=type^{i}\left(n\right)\right)$ checks whether the types of nodes in test tree and trained model are the same or not:

(20) $$p\left(\omega^{⋆}\left(n\right)|type=type^{i}\left(n\right)\right)=\left\{\begin{array}{cc} 1 & if\;type^{⋆}=type^{i}\left(n\right)\\ 0 & otherwise. \end{array}\right.$$

$p\left(duration^{⋆}\left(n\right)|{∆}_{duration}^{i}\left(n\right)\right)$ measures the difference between activity instance $\omega^{⋆}$’s duration and activity model *i* bounded between 0 and 1.

(21) $$\begin{array}{cc} p\left(\omega_{⋆}\left(n\right)|\mu =\mu_{duration}^{i}\left(n\right)\right) & \propto \exp^{-Dist_{duration}\left(n\right)}\\ \mathrm{where}\\ Dist_{duration}\left(n\right) & =\frac{|duration^{⋆}\left(n\right)-\mu_{duration}^{⋆}\left(n\right)|}{\sigma^{i}}. \end{array}$$

$p\left(\omega^{⋆}\left(n\right)|l=Distance^{i}\left(n\right)\right)$ compares the distance of training node’s trained codebooks *V* and the test node’s computed descriptor histogram *H*.

(22) $$\begin{array}{c} p\left(\omega^{⋆}\left(n\right)|l=Distance^{i}\left(n\right)\right)=\left\{\begin{array}{cc} 1 & if\;Distance{\left(H,V\right)}^{⋆}\left(n\right)=min(Distance^{i}\left(n\right)\\ 0 & otherwise. \end{array}\right. \end{array}$$

It should be noted that the Distance information is only available at root level l=0 (only for DAs). The recursion stops when it traverses all the leaves (exact inference). Once we computed $p(\omega^{⋆}|\Omega )$ for all model assignments, using MAP estimation, the activity model *i* that maximizes the likelihood function $p(\omega_{i}|\omega^{⋆})$ votes for the final recognized activity label (Equation (23)).

(23) $$\hat{i}=\arg\max_{i}\tilde{p}\left(\omega^{⋆}|\omega_{i}\right).$$

## 4. Experiments and Discussion

### 4.1. Datasets

The performance of the proposed framework is evaluated on two public and one private daily living activity datasets.

#### 4.1.1. GAADRD Dataset

**Figure 7:**
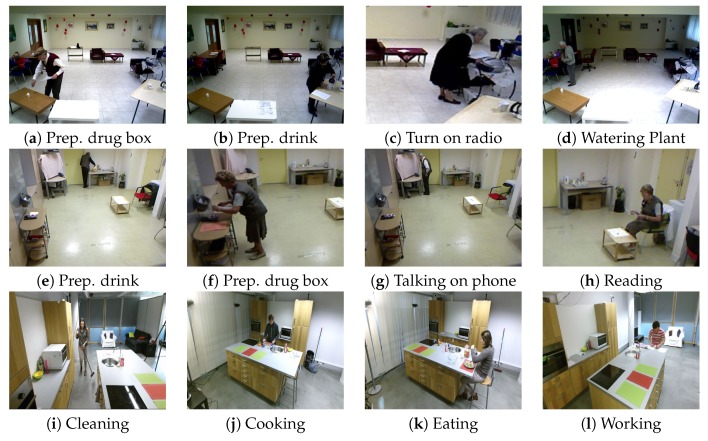
(**a**–**d**) Instances of daily activities provided in GAADRD; (**e**–**h**) CHU; and (**i**–**l**) DAHLIA datasets.

The GAADRD [84] activity dataset consists of 25 people with dementia and mild cognitive impairment who perform ADLs in an environment similar to a nursing home. The GAADRD dataset is public and was recorded under the EU FP7 Dem@Care Project (http://www.demcare.eu/results/datasets) in a clinic in Thessaloniki, Greece. The camera monitors a whole room where a person performs directed ADLs. The observed ADLs include: “Answer the Phone”, “Establish Account Balance”, “Prepare Drink”, “Prepare Drug Box”, “Water Plant”, “Read Article”, “Turn On Radio”. A sample of images for each activity is presented in Figure 7 (top row). Each person is recorded using an RGBD camera of 640×480 pixels of resolution. Each video lasts approximately 10–15 min. We randomly selected 2/3 of the videos for training and the remaining for testing.

#### 4.1.2. CHU Dataset

This dataset is recorded in the Centre Hospitalier Universitaire de Nice (CHU) in Nice, France. The hospitals collecting the dataset have obtained the agreement of an ethical committee. Volunteers and their carers have signed informed consent. Data have been anonymized and can be used only for research. It contains videos from patients performing everyday activities in a hospital observation room. The activities recorded for this dataset are “Prepare Drink”, “Answer the Phone”, “Reading Article”, “Watering Plant”, “Prepare Drug Box”, and “Checking Bus Map”. A sample of images for each activity is illustrated in Figure 7 (middle row). Each person is recorded using an RGBD Kinect camera with 640 pixels × 480 pixels of resolution, mounted on the top corner of the room. The hospital dataset is recorded under the EU FP7 DemCare project (https://team.inria.fr/stars/demcare-chu-dataset/) and it contains 27 videos. For each person, the video recording lasts approximately 15 min. Domain experts annotated each video regarding the ADLs. Similar to GAADRD, for this dataset, we randomly chose 2/3 of the videos for training and the rest for testing.

#### 4.1.3. DAHLIA Dataset

The DAHLIA dataset [85] consists of a total of 153 long-term videos of daily living activities (51 videos recorded from three different views) from 44 people. The average duration of the videos is 39 min containing 7 different actions (and a Neutral class). The considered ADLs are: “Cooking”, “Laying Table”, “Eating”, “Clearing Table”, “Washing Dishes”, “Housework”, and “Working” (Figure 7i–l). To evaluate this dataset, we followed a cross-subject protocol in order to compare our results with existing literature.

### 4.2. Evaluation Metrics

We use various evaluation metrics on each dataset to evaluate our results and compare it with other approaches. For the GAADRD and CHU datasets, we use Precision and recall metrics. True Positive Rate (TPR) or recall is the proportion of actual positives which are identified correctly: $TPR=\frac{TP}{TP+FN}$. The higher the value of this metric, the better is the performance. Similarly, Positive Predictive Value (PPV) or precision is defined as: $PPV=\frac{TP}{TP+FP}$. We also use F-score in our comparisons. The detected intervals are compared against the ground-truth intervals and an overlap higher than 80% of the ground-truth interval is considered as a True Positive detection of that activity.

For evaluation of the unsupervised framework, as the recognized activities are not labeled, there is no matching ground-truth activity label for them. The recognized activities are labeled, such as “Activity 2 in Zone 1”. In order to evaluate the recognition performance, first, we map the recognized activity intervals on the labeled ground-truth ranges. Next, we evaluate the one-to-one correspondence between a recognized activity and a ground-truth label. For example, we check which ground-truth activity label co-occurs the most with “Activity 2 in Zone 1”. We observe that in 80% of the time, this activity coincides with “Prepare Drink” label in the ground-truth. We, therefore, infer that “Activity 2 in Zone 1” represents “Prepare Drink” activity. For this purpose, we create a correspondence matrix for each activity which is defined as a square matrix where its rows are the recognized activities and the columns are ground-truth labels. Each element of the matrix shows the number of co-occurrences of that recognized activity with the related ground-truth label in that column:

$$COR\left(RA,GT\right)=\left[\begin{array}{ccccc} a_{11} & a_{12} & a_{13} & \cdots & a_{1n}\\ a_{21} & a_{22} & a_{23} & \cdots & a_{2n}\\ \vdots & \vdots & \vdots & \ddots & \vdots \\ a_{n1} & a_{n2} & a_{n3} & \cdots & a_{nn}. \end{array}\right]$$

$a_{ij}\in Z^{+}$ shows the correspondence between activity instance *i* and ground-truth label *j*. RA is the set of recognized activity instances and GT shows the set of ground-truth labels. We evaluate the performance of the framework based on the inferred labels. These labels are used for calculating the *Precision*, *Recall*, and *F-Score* metrics.

In order to evaluate the DAHLIA dataset, we use metrics based on frame level accuracy. For each class *c* in the dataset, we assume ${TP}^{c},{FP}^{c},{TN}^{c}$, and $FN^{c}$ as the number of True Positive, False Positive, True Negative, and False Negative frames, respectively. Therefore, Frame-wise accuracy is defined as: $FA_{1}=\frac{\sum_{c\in C}TP^{c}}{\sum_{c\in C}N_{c}}$, where $N_{c}$ is the number of correctly labeled frames compared to the ground-truth. F-Score is defined as: $F-Score=\frac{2}{\left|C\right|}\sum_{c\in C}\frac{P^{c}\times R^{c}}{P^{c}+R^{c}}$ where $P^{c}$ and $R^{c}$ are precision and recall metrics of class *c*, respectively. We also define Intersection over Union (IoU) metric as:

(24) $$IoU=\frac{1}{\left|C\right|}\sum_{c\in C}\frac{TP^{c}}{TP^{c}+FP^{c}+FN^{c}}.$$

*C* is the total number of action classes.

### 4.3. Results and Discussion

**Figure 8:**
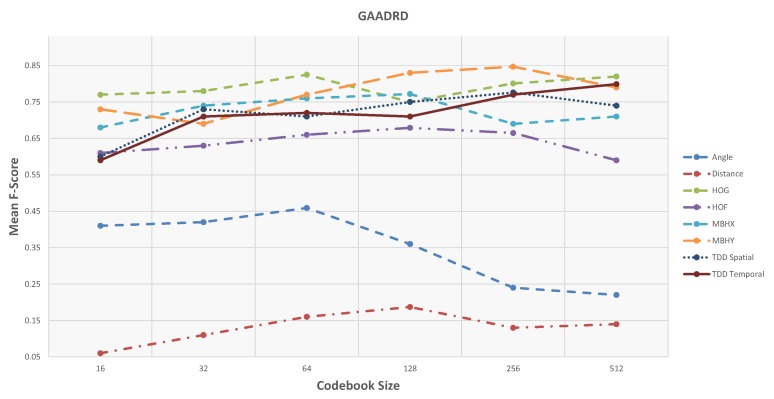
Shows F-Score values of the unsupervised framework w.r.t. codebook size on GAADRD dataset.

First, the results and evaluations of the three datasets are reported and then compared with state-of-the-art methods. Different codebook sizes are examined for the Fisher vector dictionaries: 16, 32, 64, 128, 256, and 512. Table 1 and Figure 8 show the accuracy of activity detection based on Precision and Recall metrics using the feature type with the highest accuracy. In the case of the GAADRD dataset, the best result achieved with incorporated Motion Boundaries Histogram in *Y* axis (MBHY) descriptor in the activity models with codebook size set to 256.

Based on the obtained results, there is no special trend regarding the codebook size. For some features (MBHY and TDD spatial), the performance increases with an increase in the codebook size and drops when the codebook size becomes much bigger. For TDD temporal feature, performance increases linearly with the codebook size. For the geometrical features, particularly for the Angle feature, there is a big drop of performance with bigger codebook sizes. For others (HOG, HOF), medium-size codebook performs the best. Finding an optimal codebook size is challenging. Small datasets usually work better with smaller codebook size, and as the datasets’ size grows, the codebook performs better. Regardless of the codebook size, MBHY descriptor performs better than other features in this dataset. The MBH descriptor is composed of *X* (MBHX) and *Y* (MBHY) components. As the activities involve many vertical motions, MBHY descriptor is able to model the activities better compared to the other dense trajectory descriptors and even deep features. It can be noticed that the performance of temporal deep features gets better as the codebook size gets bigger. In addition, motion features (TDD temporal, MBHY) perform better than appearance features and temporal deep features perform better than spatial TDDs. The reason for the lower performance of appearance features might be due to the activities performed in a hospital environment. Hereupon, the background does not contain discriminative information which can be encoded in activity models. It is clear that the Geometrical features perform poorly. Daily living activities are comprised of many sub-activities with similar motion patterns related to object interactions. It seems that geometrical features do not contain sufficient information to ensure encoding these interactions which result in poor detection. Furthermore, the confusion matrix in Figure 10 indicates that the activities with similar motion in their sub-activities are confused with each other the most.

**Figure 9:**
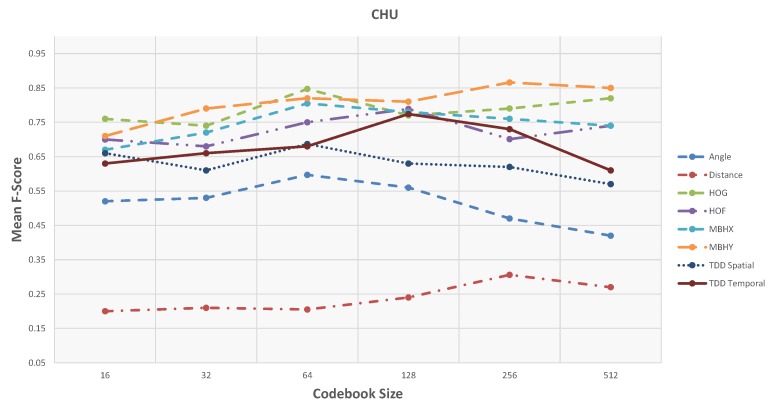
Shows F-Score values of the unsupervised framework w.r.t. codebook size on CHU dataset.

**Figure 10:**
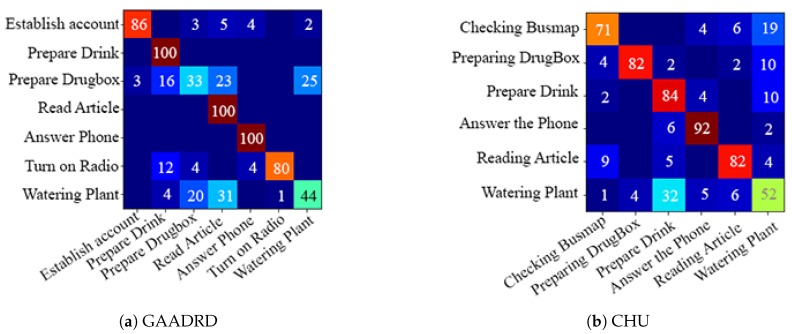
Confusion matrices regarding the best configuration of the unsupervised framework on (**a**) GAADRD and (**b**) CHU datasets (with Motion Boundaries Histogram in *Y* axis (MBHY) descriptor). The values show mean accuracy (%).

On CHU dataset, the unsupervised framework achieves promising results (Table 2 and Figure 9). Similar to the GAADRD dataset, the effect of codebook size is different for different descriptor types. For MBHY descriptor, the accuracy increases as codebook size grow, whilst, it has the opposite effect on TDD appearance features. Differently, the accuracy increases and then decreases for TDD temporal feature. It can be observed that a bigger codebook size results in better performance. This trend is different from GAADRD dataset and the reason might be because of the larger size of this dataset. TDD temporal features demonstrate a better performance than deep appearance features (TDD spatial). Similarly, due to the similar background of the activities, temporal information shows better results. MBHY achieves the best performance on this dataset. The abundance of vertical motions in the performed activities helps the MBH descriptors to reach better recognition performance. Among appearance features, HOG descriptor shows a better performance since it can encode the appearance information efficiently, where it even outperforms deep appearance features. Detailed analysis (Figure 10) indicates that the framework has difficulty in recognition of “Watering Plant“ activity. It confuses this activity with all the other activities. The short duration of this activity leads to insufficient capture of local dynamic information resulting in recognition issues. The reason for the confusion of the other activities lies mainly on similar motion patterns of the sub-activities. Moreover, this dataset consists of activities recorded from subjects lateral view which makes recognition of those classes of activities challenging.

### 4.4. Comparisons

This section summarizes the evaluations and comparisons conducted on GAADRD (Section 4.5), CHU (Section 4.6), and DAHLIA (Section 4.7) datasets.

The results obtained from our proposed framework on GAADRD and CHU datasets are compared with the supervised approach in [75], where videos are manually clipped. Another comparison is made with an online supervised approach that follows [75] using a sliding window scheme. The activity models are evaluated with another version of the models [86] that does not embed local dynamic information (in this version, the score of the local descriptor attribute is omitted and not considered in the final score). A further comparison is performed with a Hybrid framework [87] that combines supervised and unsupervised information in the HAM models. We additionally compare GAADRD dataset with the produced results of another detection algorithm in [88].

### 4.5. GAADRD Dataset

Table 3 represents the comparison of our results with the reported performance on GAADRD dataset. In all approaches that use body motion and appearance features, the feature types with the best performances are selected. It can be noticed that using models equipped with both global and local motion features, the unsupervised obtains high sensitivity and precision rates. Compared to the online version of [75], thanks to the learned zones and discovered activities, we obtain better activity localization, thereby a better precision. Using only dense trajectories (not global motion) this online method fails to localize activities. For the “Watering Plant” this method can not detect any instances of this activity in the test set, hence, the Precision, Recall, and F-Score rates are zero. Compared to the unsupervised approach that either uses global motion features or body motion features, we can see that, by combining both features, our approach achieves more discriminative and precise models and improves both sensitivity and precision rates. Although the supervised approach in [75] outperforms the unsupervised framework in recall and F-Score metrics, it actually does not perform activity detection. It uses ground-truth intervals provided by manual clipping and performs offline activity recognition which is a much simpler task. As our approach learns the scene regions, we automatically discover the places where the activities occur, thereby we achieve precise and accurate spatiotemporal localization with a lower cost. As scene region information is missing in the supervised approach, it detects “Turning On Radio” while the person is inside the “Preparing Drink” region. On this dataset, the unsupervised method always performs better than the “Online Supervised” approach and significantly outperforms the sequential statistical boundary detection (SSBD) method. It also outperforms another unsupervised version of the framework while no descriptor information is used in the activity models. Only the supervised methods surpass our unsupervised models. The reason is that the supervised method works with pre-clipped activity videos and overlooks the challenging task of temporal segmentation of activity samples from the original video flow.

### 4.6. CHU Dataset

Table 4 shows the results of evaluated approaches and their comparison with our results on CHU Nice Hospital dataset. In this dataset, as people tend to perform some of the activities in various regions (e.g., preparing the drink at the phone desk), it is difficult to obtain high precision rates. However, compared to the online version of the supervised method in [75], our approach detects all activities and achieves a much better precision rate. The online version of [75] again fails to detect activities accurately and misses some of the “Prepare Drink”, and “Reading Article“ activities and produces lots of false positives for all other activities. It cannot handle the transition states in the boundary of the activity regions (e.g., walking from telephone desk to DrugBox is detected as “Answer the Phone“ activity). For this reason, a random label is assigned for transition states by the classifier, which consequently increases the rate of false positives. Compared to the Online Supervised method, we have increased the average precision rate from 48.06 to 87.65%. Compared to the unsupervised method without embedded descriptor information, we have decreased the false positive rates and increased the precision rates significantly. The highest improvements are on “Answering Phone“ from 60 to 92%, “Checking BusMap“ from 54.54 to 80.5%, “Prepare Drink“ from 80 to 94%, and “Watering Plant“ from 53 to 77%. For “Reading Article“ activity, there is a small increase in false positive rates, causing an incremental decrease in precision rates. This might be because of the lack of local motion information caused by staying still in a sitting posture for a long time. Since the motion representation of [86] contains only global information, it fails to distinguish activities inside the regions precisely. For instance, passing by the phone zone and answering the phone in the phone zone are considered as the same activity in their models. Hence, their unsupervised approach results in high false positive rates. In addition, we can observe that the proposed approach improves the true positive rates and increased sensitivity rates for most of the activities when it is compared to the “Only Global Motion“ method.

### 4.7. DAHLIA Dataset

Different from the two other datasets, the results on the DAHLIA dataset are compared with all the previous evaluations we could find in the literature. Meshry et al. [89] exploits gesturelets extracted from skeleton data to compute geometrical features and detect the activities. The proposed method in [90] takes a graphical approach and poses the activity detection task as a maximum-weight connected sub-graph problem. Inspired by the Hough transformation that is successfully applied in object detection, Chan-Hon-Tong et al. [91] proposes a method with discriminative features to globally optimize the parameters of Hough transform and utilize it for activity segmentation in videos. Finally, our results are compared with [92] that is a supervised method with a semi-supervised component to discover sub-activities. Table 5 demonstrates our results on the DAHLIA dataset. Different metrics are used for evaluation of this dataset to enable comparison with other methods. The table presents the best results that are produced by the generated models embedded with MBHY descriptors. It can be noticed that in this dataset, we significantly outperform [89,90] in all the categories. Efficient Linear Search (ELS) uses geometrical features and produces poor results that are only comparable with our framework when geometrical descriptors are used in the generated models. Despite being an efficient approach, Chen and Grauman [90] demonstrates poor detection performance on Dahlia dataset. Additionally, this method only works in offline mode. Chan-Hon-Tong et al. [91] is another supervised method that uses both skeleton and dense trajectory descriptors and outperforms our framework only on camera view 3, while using the F-score metric. The closest performance to ours is [92] which is a supervised method and utilizes person-centered CNN features (PC-CNN) to detect sub-activities. Moreover, it has an additional post-processing step to refine the sub-activity proposals in the activity boundaries. Although our framework is totally unsupervised, we outperform this method in camera view 2 using all evaluation metrics. Similar results are obtained using different camera angles underlying the robustness of our proposed framework to viewpoint variations and different types of occlusion. This indicates that an efficient multi-view fusion method can remarkably improve the results.

Overall, although our unsupervised framework does not utilize any supervised information, it achieved promising recognition performances. Compared to the fully supervised hybrid method [87], the unsupervised framework obtains acceptable and competitive results in the detection of most of the activities. However, the high performance of the hybrid method comes with the cost of human supervision. In the hybrid method, a supervised Support Vector Machine (SVM) classifier is trained with the ground-truth annotation provided by a human. The main benefits of the unsupervised method are automatic online clipping and detection of activities as well as unsupervised modeling and recognition. With all these benefits, the marginal difference in the recognition rate of the unsupervised method relative to supervised counterparts is admissible.

## 5. Conclusions

An online unsupervised framework is proposed for detection of daily living activities, particularly for elderly monitoring. To create the activity models, we benefited from the superiority of unsupervised approaches on representing global motion patterns. Then, discriminative local motion features were employed in order to generate a more accurate model of activity dynamics. Thanks to the proposed scene model, online recognition of activities can be performed with reduced user interaction for clipping and labeling a huge amount of short-term actions which are essential for most of the previously proposed methods. Our extensive evaluations on three datasets revealed that our proposed framework is capable of detecting and recognizing activities in challenging scenarios. The evaluations were intentionally conducted on the datasets recorded in nursing homes, hospitals, and smart homes to examine the implication of the method on ambient surveillance in such environments. Further work will investigate how to generate generic models that can detect activities in any environment with minimum modification of the models. Our goal is to use the developed framework in the evaluation of long-term video recordings in nursing homes and to assess the performance of the subjects to impose early interventions which will result in early diagnosis of cognitive disorders, especially Alzheimer’s disease.

## Figures and Tables

**Table 1:** Results related to the unsupervised framework with different feature types on GAADRD dataset.

	32			64			128			256			512		
	Prec. (%)	Rec. (%)	F-Score	Prec. (%)	Rec. (%)	F-Score	Prec. (%)	Rec. (%)	F-Score	Prec. (%)	Rec. (%)	F-Score	Prec. (%)	Rec. (%)	F-Score
Angle	57.6	33.2	0.42	61.2	36.1	0.45	46.9	30.2	0.36	28.1	22.4	0.24	26.7	19.8	0.22
Distance	12.9	9.7	0.11	18.2	14.9	0.16	20.7	16.1	0.18	14.7	12.1	0.13	14.7	15.2	0.14
HOG	81.4	75.2	0.78	84.7	79.6	0.825	77.5	74.3	0.75	82.7	77.6	0.80	84.7	79.8	0.82
HOF	64.6	61.9	0.63	64.9	67.7	0.66	66.1	68.1	0.67	65.4	67.9	0.66	57.4	62.1	0.59
MBHX	71.3	77.2	0.74	74.8	78.2	0.76	79.8	76.1	0.77	67.6	72.1	0.69	69.4	72.8	0.71
MBHY	71.5	68.4	0.69	78.8	76.1	0.77	82.7	84.9	0.83	83.1	85.7	0.84	80.2	79.4	0.79
TDD Spatial	74.5	72.9	0.73	72.8	71.2	0.71	77.5	74.3	0.75	77.5	76.9	0.77	76.4	73.5	0.74
TDD Temporal	73.4	69.1	0.71	73.9	70.6	0.72	72.5	69.9	0.71	79.4	76.2	0.77	81.9	76.9	0.79

**Table 2:** Results regarding the unsupervised framework with different feature types on CHU dataset.

	32			64			128			256			512		
	Prec. (%)	Rec. (%)	F-Score	Prec. (%)	Rec. (%)	F-Score	Prec. (%)	Rec. (%)	F-Score	Prec. (%)	Rec. (%)	F-Score	Prec. (%)	Rec. (%)	F-Score
Angle	58.4	49.7	0.53	60.7	57.8	0.59	58.6	55.2	0.56	50.3	45.9	0.47	41.7	44.1	0.42
Distance	23.9	19.2	0.21	22.7	19.5	0.20	27.8	21.7	0.24	29.2	31.9	0.30	28.8	27.1	0.27
HOG	77.7	71.9	0.74	85.7	82.9	0.84	80.8	74.9	0.77	81.9	76.3	0.79	84.9	79.8	0.82
HOF	68.2	69.8	0.68	73.9	76.4	0.75	77.1	79.1	0.78	68.4	71.9	0.70	73.4	74.9	0.74
MBHX	73.4	72.1	0.72	81.3	80.4	0.80	78.6	79.2	0.78	75.2	78.3	0.76	73.4	76.2	0.74
MBHY	80.5	77.9	0.79	84.3	79.9	0.82	83.9	79.3	0.81	88.6	83.6	0.866	87.4	83.1	0.85
TDD Spatial	65.8	58.4	0.61	71.9	64.7	0.68	67.2	60.9	0.63	65.9	60.1	0.62	60.0	55.9	0.57
TDD Temporal	67.7	65.7	0.66	69.7	66.1	0.68	79.2	76.1	0.77	74.4	73.5	0.73	61.8	62.1	0.61

**Table 3:** Comparison of different recognition frameworks with ours on the GAADRD dataset. The diagram shows the class-wise accuracy of each method with respect to their F-Score values. The best results in each section are indicated in bold.

	Supervised (Manual Clipping) with HOG, Dict sz = 512 [75]			Online Version of [75]			Classification by Detection SSBD [88]			Unsupervised Using Only Global Motion [86]			Hybrid [87]			Unsupervised (Proposed Method)		
	Prec. (%)	Rec. (%)	F-Score	Prec. (%)	Rec. (%)	F-Score	Prec. (%)	Rec. (%)	F-Score	Prec. (%)	Rec. (%)	F-Score	Prec. (%)	Rec. (%)	F-Score	Prec. (%)	Rec. (%)	F-Score
Establish Account	92.2	84.3	0.88	29.1	**100**	0.45	41.67	41.67	0.41	86.2	**100**	0.92	**92.3**	**100**	**0.95**	86.2	**100**	0.92
Prepare Drink	92.1	**100**	0.95	69.4	**100**	0.81	80.0	96.2	0.87	**100**	78.1	0.87	**100**	92.1	0.95	**100**	100	**1.0**
Prepare DrugBox	94.9	85.5	**0.89**	20.2	11.7	0.14	51.28	86.96	0.64	**100**	33.34	0.50	78.5	**91.3**	0.84	**100**	33.1	0.49
Reading Article	96.2	96.2	0.96	37.8	88.6	0.52	31.88	**100**	0.48	**100**	**100**	1.0	**100**	**100**	**1.0**	**100**	**100**	**1.0**
Answer the Phone	88.5	**100**	0.93	70.1	**100**	0.82	34.29	96.0	0.50	**100**	**100**	**1.0**	**100**	91.2	0.95	**100**	**100**	**1.0**
Turn On Radio	**89.4**	86.7	0.88	75.1	**100**	0.85	19.86	96.55	0.32	89.0	89.0	0.89	89.1	93.4	**0.91**	89.1	89.3	0.89
Watering Plant	84.8	72.6	0.78	0	0	0	44.45	**86.36**	0.58	57.1	44.45	0.49	79.9	86.1	0.82	**100**	44.2	0.61
Average	91.16	89.33	0.90	43.1	71.4	0.51	43.34	86.24	0.54	90.32	77.84	0.81	91.4	**93.44**	**0.92**	**96.47**	80.94	0.84

**Table 4:** Comparison of different recognition frameworks with ours on the CHU dataset. The table below shows the detailed results of each method with respect to each class in the dataset. The best results in each section are indicated in bold.

	Supervised (Manual Clipping) with HOG, Dict sz = 256 [75]			Online Version of [75]			Unsupervised Using Only Global Motion [86]			Hybrid			Unsupervised (Proposed Method)		
	Prec. (%)	Rec. (%)	F-Score	Prec. (%)	Rec. (%)	F-Score	Prec. (%)	Rec. (%)	F-Score	Prec. (%)	Rec. (%)	F-Score	Prec. (%)	Rec. (%)	F-Score
Checking BusMap	**100**	97.1	**0.98**	50.1	**100**	0.66	54.54	**100**	0.70	96.1	**100**	**0.98**	80.5	86.2	0.83
Prepare DrugBox	**100**	92.3	0.95	43.2	**100**	0.60	**100**	90.1	0.94	**100**	**100**	**1.0**	88.2	92.7	0.90
Prepare Drink	**93.1**	**97.4**	**0.95**	38.1	76.1	0.50	80.0	84.21	0.82	88.9	96.3	0.92	**94.2**	88.5	0.91
Answer the Phone	92.2	**100**	0.95	86.7	**100**	0.92	60.1	**100**	0.75	**100**	**100**	**1.0**	92.4	**100**	0.96
Reading Article	97.5	94.1	0.95	36.4	92.0	0.52	**100**	81.82	0.90	**100**	**100**	**1.0**	93.2	87.4	0.90
Watering Plant	**100**	88.3	**0.93**	33.9	76.9	0.47	53.9	68.9	0.60	77.0	96.3	0.85	77.4	61.2	0.68
Average	**97.13**	94.87	0.95	48.06	90.83	0.61	74.75	87.50	0.78	93.66	**98.76**	**0.96**	87.65	86.00	0.86

**Table 5:** The activity detection results obtained on the DAHLIA. Values in bold represent the best performance.

	ELS [89]			Max Subgraph Search [90]			DOHT (HOG) [91]			Sub Activity [92]			Unsupervised (proposed method)		
	FA_1	F_score	IoU	FA_1	F_score	IoU	FA_1	F_score	IoU	FA_1	F_score	IoU	FA_1	F_score	IoU
**View 1**	0.18	0.18	0.11	-	0.25	0.15	0.80	0.77	0.64	**0.85**	**0.81**	**0.73**	0.84	0.79	0.70
**View 2**	0.27	0.26	0.16	-	0.18	0.10	0.81	0.79	0.66	0.87	0.82	0.75	**0.88**	**0.83**	**0.77**
**View 3**	0.52	0.55	0.39	-	0.44	0.31	0.80	**0.77**	0.65	**0.82**	0.76	**0.69**	0.79	0.73	**0.69**

## Abbreviations

The following abbreviations are used in this manuscript:

ADL	Activities of Daily Living
CNN	Convolutional Neural Networks
RNN	Recurrent Neural Network
LSTM	Long Short-Term Memory
C3D	Convolution3D
TCN	Temporal Convolutional Network
HDP	Hierarchical Dirichlet Process
HOG	Histogram of Oriented Gradients
HOF	Histogram of Optical Flow
MBH	Motion Boundaries Histogram
MBHX	Motion Boundaries Histogram in X axis
MBHY	Motion Boundaries Histogram in Y axis
TSD	Trajectory Shape Descriptor
TDD	Trajectory-Pooled Deep-Convolutional Descriptors
BIC	Bayesian Information Criterion
SR	Scene Region
PE	Primitive Event
DA	Discovered Activity
FV	Fisher Vector
HAM	Hierarchical Activity Model
MAP	Maximum A Posteriori
TP	True Positive
FP	False Positive
TN	True Negative
FN	False Negative
TPR	True Positive Rate
PPV	Positive Predictive Value
IoU	Intersection over Union
SSBD	Sequential statistical boundary detection
ELS	Efficient Linear Search
PC-CNN	Person-Centered CNN
SVM	Support Vector Machine
